# Role of Serotonin Neurons in L-DOPA- and Graft-Induced Dyskinesia in a Rat Model of Parkinson's Disease

**DOI:** 10.1155/2012/370190

**Published:** 2012-06-11

**Authors:** Eunju Shin, Elisabetta Tronci, Manolo Carta

**Affiliations:** ^1^Division of Neurobiology, Wallenberg Neuroscience Center, Lund University, 221 84 Lund, Sweden; ^2^Department of Biomedical Science, Cagliari University, Cittadella Universitaria, SS 554 km 4.500, 09042 Monserrato, Italy

## Abstract

L-DOPA, the most effective drug to treat motor symptoms of Parkinson's disease, causes abnormal involuntary movements, limiting its use in advanced stages of the disease. An increasing body of evidence points to the serotonin system as a key player in the appearance of L-DOPA-induced dyskinesia (LID). In fact, exogenously administered L-DOPA can be taken up by serotonin neurons, converted to dopamine and released as a false transmitter, contributing to pulsatile stimulation of striatal dopamine receptors. Accordingly, destruction of serotonin fibers or silencing serotonin neurons by serotonin agonists could counteract LID in animal models. Recent clinical work has also shown that serotonin neurons are present in the caudate/putamen of patients grafted with embryonic ventral mesencephalic cells, producing intense serotonin hyperinnervation. These patients experience graft-induced dyskinesia (GID), a type of dyskinesia phenotypically similar to the one induced by L-DOPA but independent from its administration. Interestingly, the 5-HT_1A_ receptor agonist buspirone has been shown to suppress GID in these patients, suggesting that serotonin neurons might be involved in the etiology of GID as for LID. In this paper we will discuss the experimental and clinical evidence supporting the involvement of the serotonin system in both LID and GID.

## 1. Introduction

Parkinson's disease (PD) is the second most common neurodegenerative disease and is characterized by loss of dopamine (DA) neurons in the substantia nigra. The cell loss results in decreased activation of striatal DA receptors, thus causing motor impairments, such as tremor, rigidity, bradykinesia, and postural instability. The DA precursor L-3,4-dihydroxyphenylalanine (L-DOPA) represents the most effective drug to alleviate the motor symptoms. Although this medication is very efficient during the first few years of administration, its efficacy gradually diminishes overtime, and uncontrolled excessive movements, known as dyskinesia, appear as a side effect after a variable number of years in most of patients, limiting the use of L-DOPA in advanced stages of the disease. 

A better understanding of the mechanisms underlying the appearance of dyskinesia has been achieved during recent years using animal models of L-DOPA-induced dyskinesia (LID). In fact, abnormal involuntary movements (AIMs) develop in response to sub-chronic L-DOPA treatment in 6-hydroxydopamine (6-OHDA)-lesioned rats and 1-methyl-4-phenyl-1,2,3,6-tetrahydropyridine (MPTP)-treated monkeys, resembling peak-dose dyskinesia seen in patients [1–4]. Using these models, a number of alterations have been identified at the level of striatal neurons of dyskinetic subjects, such as abnormal trafficking of DA D_1_ and *N*-methyl-D-aspartate (NMDA) receptors [5, 6], leading to alterations in key striatal signaling pathways. 

More recently, the serotonin system has emerged as a putative player in the induction of fluctuations in synaptic DA levels following administration of L-DOPA in animal models of PD, which cause pulsatile stimulation of DA receptors and promote the maladaptive changes that characterize the parkinsonian dyskinetic brain [7–11]. The emerging role of serotonin neurons in LID has also prompted researchers to investigate a possible involvement of these neurons in the appearance of *off-drug* dyskinesia that has emerged in a subset of PD patients following transplantation of fetal ventral mesencephalic DA neuroblasts, namely, graft-induced dyskinesia (GID) [12, 13]. In this paper, we will discuss the recent experimental and clinical evidence supporting a role of serotonin neurons both in LID and GID.

## 2. The Role of Serotonin Neurons in the Induction of L-DOPA-Induced Dyskinesia

Progression of DA neuron degeneration represents the first risk factor for development of LID in patients. In fact, the efficacy of L-DOPA in providing its therapeutic effect during the first years of administration is conceivably due to the ability of spared DA neurons to take up exogenously administered L-DOPA, convert it to DA, and release it into the synaptic cleft, but also to regulate synaptic DA levels via the D_2_ autoreceptor and DA transporter (DAT). When the disease is in its early stage, sufficient DA terminals remain in the striatum to efficiently mediate a feedback-controlled mechanism of release. The ability of the spared DA terminals to prevent development of AIMs upon L-DOPA treatment is well demonstrated in a recent report by Ulusoy et al. [14]. In this study, a significant DA deficiency was established in rats by viral vector delivery of short hairpin RNA for the tyrosine hydroxylase (TH) enzyme, without affecting cell survival. Animals were then made dyskinetic by subchronic treatment with the direct DA agonist apomorphine; however, when treated with L-DOPA, the same rats appeared to be fully resistant to the induction of LID, despite apomorphine treatment had already promoted postsynaptic alterations, such as increased striatal FosB expression.

The ability of the presynaptic DA compartment to prevent excessive DA receptor stimulation, and aberrant downstream signaling, even in presence of supersensitive striatal DA receptors is also confirmed in transplantation studies. In fact, ventral mesencephalic DA graft, which reconstitutes the presynaptic buffering capacity into the lesioned and maladapted striatum, normalizes the response of L-DOPA-primed dyskinetic rats to L-DOPA administration [7]. In light with these results, it is conceivable to think that the efficacy of L-DOPA during the first years of administration is also due to the presence of a sufficient number of DA neurons that can buffer the exogenously administered L-DOPA, and provide a source of regulated DA release. However, with the progression of DA degeneration, this buffering capacity, and thus the ability to provide physiological level of DA receptor stimulation, is progressively lost. In this situation, the serotonin neurons come to play a major role in L-DOPA-derived DA production and release, as they possess both the aromatic amino acid decarboxylase enzyme (AADC) and the vesicular monoamine transporter (VMAT). Unlike DA neurons, though, serotonin neurons cannot regulate the extracellular levels of DA due to the lack of the autoregulatory loop, hence, causing un-controlled DA release following L-DOPA administration. DA released from serotonin neurons will therefore act in concert with the intermittent nature of the orally administered L-DOPA to cause pulsatile stimulation of DA receptors, and thus, changes in downstream signaling pathways at striatal neurons. In support of this view, removal of serotonin innervation by toxin lesion was shown to produce about 80% reduction in L-DOPA-derived striatal extracellular DA levels [15], and to induce a near-to-complete suppression of LID in dyskinetic rats [8, 16]. Accordingly, pharmacological silencing of serotonin neuron activity by 5-HT_1A_ and 5-HT_1B_ receptor agonists has been shown to reduce L-DOPA-derived extracellular DA levels [17], and to suppress LID in rats [8] as well as in MPTP-treated monkeys [18]. In addition, chronic administration of the 5-HT_1_ agonists was able to prevent the development of dyskinesia and upregulation of FosB expression in the striatum of 6-OHDA-lesioned rats [18], thus linking dysregulated DA release from serotonin terminals with the induction of a well-known striatal marker of dyskinesia. Interestingly, simultaneous activation of 5-HT_1A_ and 5-HT_1B_ receptors was found to trigger a potent synergistic effect in the suppression of LID in both rats and monkeys [8, 18]. In fact, LID was nearly fully abolished at doses of combined agonists that were ineffective when given individually. This finding has now led to the initiation of a first double-blind, proof-of-concept clinical trial employing a mixed 5-HT_1A/1B_ receptor agonist in dyskinetic patients. 

In confirming the interaction between exogenously administered L-DOPA and serotonin neurons, Navailles and coworkers have recently demonstrated that serotonin neuron-dependent DA release takes place, upon chronic L-DOPA treatment in 6-OHDA-lesioned rats, not only in the striatum but also in other brain areas receiving sufficient serotonin innervation, such as substantia nigra, hippocampus, and prefrontal cortex [19]. Moreover, L-DOPA administration appears to result in reduced striatal serotonin tissue content in 6-OHDA-lesioned rats [8]. The latter result supports the existence of a competition between serotonin and DA for serotonergic vesicles, which causes serotonin depletion. Thus, an increasing body of experimental evidence points to the serotonin system as a key player in the appearance of LID. 

Progressive reduction of L-DOPA-derived extracellular DA levels upon chronic L-DOPA treatment has been recently found in 6-OHDA-lesioned rats [20, 21], leading some researcher to question the role of serotonin neurons in the appearance of LID [21]. Nevertheless, we believe that the potent inhibitory effect of 5,7-dihydroxytryptamine (5,7-DHT) lesion on both development and expression of dyskinesia in 6-OHDA-lesioned rats [8], together with the striking suppression of LID induced by low doses of 5-HT_1A_ + 5-HT_1B_ receptor agonists both in rats and macaques [18] provided unquestionable evidence supporting an important role of serotonin neurons, at least in animal models of PD. It should be taken into account that during chronic administration of L-DOPA, postsynaptic DA receptors become supersensitive; thus, the dyskinetic response to L-DOPA might be maintained by lower extracellular DA levels once postsynaptic alterations have been already induced. The relevance for the human disease of the progressive reduction of extracellular DA levels seen upon chronic L-DOPA in the rat 6-OHDA model remains to be established, as L-DOPA-derived synaptic DA levels were shown to increase with progression of the disease in a positron emission tomography (PET) imaging study in PD patients [22]. 

Interestingly, Rylander and coworkers have recently shown marked serotonin hyperinnervation in the lesioned striatum of parkinsonian dyskinetic subjects across different species, including patients, thus raising the possibility that serotonin neurons play a relevant role in the emergence of LID also in the patients [10, 11, 23]. This study suggested that L-DOPA treatment may be able to provoke sprouting of serotonin axon terminals and change their morphology, hence, possibly enhancing the fluctuations in extracellular DA concentration, consistent with findings of de la Fuente-Fernandez and coworkers in their PET imaging study [22, 24]. 

In further experimental support for a detrimental effect of serotonin neurons on LID, grafted serotonin neurons, which induced an intense hyperinnervation of the grafted striatum, exacerbated LID in both partial and complete DA lesioned rats [7, 25], raising the possibility that inclusion of these cells in the grafted tissue may have detrimental effects on LID in grafted PD patients. 

## 3. The Role of Serotonin Neurons in the Modulation of Graft-Induced Dyskinesia 

Transplantation of fetal ventral mesencephalic neurons is a therapeutic approach to PD that has already been tested in clinical trials [26–30]. While the variability in the outcome of these studies halted further investigations, the presence of highly responsive patients provided proof-of-concept that this therapeutic intervention can be significantly beneficial. Indeed, there is now general agreement that the reasons accounting for the observed variability rely on the lack of standardization of the cell preparations, surgical procedures, as well as on the selection of patients and presence or absence of postsurgical immunosuppressive treatment [31]. However, another element that has contributed to raise concern about fetal transplantation is the appearance in a subset of grafted patients of *off-drug* dyskinesia [29, 31–33], a form of involuntary movements that is phenotypically similar to the one induced by L-DOPA but independent from its administration. The recent findings on the role of serotonin neurons in the induction of LID has led to hypothesize that serotonin neurons included in the graft may also play a role in GID [8, 16, 18]. 

In fact, serotonin neuroblasts are located in close vicinity of the DA ones in the fetal ventral mesencephalic area that is dissected for transplantation. Accordingly, about 50% of grafted cells were found to be serotonin neurons in a post-mortem analysis of the caudate-putamen of grafted patients [34].

In possible support of this hypothesis, in a recent PET study, Politis and colleagues have found intense serotonergic hyperinnervation in the striatum of grafted patients showing GID [12, 13]. Interestingly, administration of the partial 5-HT_1A_ agonist buspirone suppressed GID in all tested patients. An involvement of the serotonin neurons in GID is further supported by the high serotonin transporter (SERT)/DAT ratio found in one GID patient compared to both healthy age-matched control and non-grafted PD patients [12]. Thus, it is postulated that serotonin terminals may take up DA released by the graft through SERT, and release DA, as a false transmitter, away from the uptake site, in striatal regions lacking sufficient DA innervation, thus, leading to activation of supersensitive DA receptors. However, it should be acknowledged that these clinical observations have been made in a very few subjects, and further evidence should be provided. In particular, it would be important to investigate the state of the striatal serotonin innervation also in patients free of GID. 

Although spontaneous GID, that is dyskinesia in the absence of any drug treatment, is inconsistent in grafted rodents [35, 36], it does appear after administration of a low dose of amphetamine [35, 37], which is known to evoke massive DA release from grafted DA neurons [38]. These abnormal movements can be scored with the same scale used for LID [35, 37], and are now widely used as a convenient and reproducible model of GID [7, 39–42]. While appearance of GID in this rat model is clearly dependent on the presence of an adequate number of DA neurons in the graft, we have recently found a bidirectional modulatory effect of endogenous serotonin neurons on GID. In fact, reduction of serotonin neuron activity, by a combination of 5-HT_1A_ and 5-HT_1B_ receptor agonists, produced a significant reduction of GID, while increased serotonin neuron release by fenfluramine exacerbated GID [43]. Strikingly, administration of a low dose of buspirone (1 mg/kg) completely suppressed GID, as seen in grafted patients. Interestingly, removal of the endogenous serotonin innervation by specific toxin lesions appeared to abolish the anti-GID effect of the selective 5-HT_1_ agonists, both in serotonin-containing and serotonin-free grafts, suggesting that the modulatory effect on GID may be due to the endogenous rather than the graft-derived serotonin neurons, at least in our experimental conditions. By contrast, neither removal of the endogenous serotonin innervation nor pretreatment with the selective 5-HT_1A_ antagonist WAY100135 reduced the anti-GID efficacy of buspirone. In fact, buspirone is also known to possess antagonistic properties on D_2_ receptors [44–47]. In support for a D_2_-mediated effect of buspirone, similar anti-GID effect was induced by a low dose of the selective D_2_ receptor antagonist eticlopride (0.03 mg/kg). Thus, our data support a modulatory role of the endogenous serotonin neurons on expression of GID, as well as a peculiar role of D_2_ receptors. Indeed, both buspirone and eticlopride were ineffective against LID at doses fully suppressing GID [43]. 

Further work is required to understand whether inclusion of serotonin neurons in the graft can be detrimental for appearance of GID, although current experimental data do not support this hypothesis. 

## 4. Conclusion

Overall, loss of DA in basal ganglia circuits and DA replacement by chronic L-DOPA administration result in complex alterations in the parkinsonian brain, that affect several systems and key signaling proteins, most of which remain poorly understood. DA released as a false transmitter from serotonin neurons appears to play a key role in initiating these events, at least in animal models. Serotonin neurons, therefore, represent an intriguing pharmacological target to treat already established LID and/or to prevent the events leading to the appearance of LID from taking place. Recent evidence suggests that serotonin neurons may also participate to the induction of dyskinesia seen in the *off-state* in grafted PD patients. The upcoming new clinical trial, funded by the European Community, employing fetal ventral mesencephalic cells will answer the question whether optimization of the surgical procedures and preparation of the grafted material, including exclusion of serotonin neuroblasts, will improve clinical outcome and avoid appearance of GID.
